# Congenital Heart Disease–Causing Gata4 Mutation Displays Functional Deficits *In Vivo*

**DOI:** 10.1371/journal.pgen.1002690

**Published:** 2012-05-10

**Authors:** Chaitali Misra, Nita Sachan, Caryn Rothrock McNally, Sara N. Koenig, Haley A. Nichols, Anuradha Guggilam, Pamela A. Lucchesi, William T. Pu, Deepak Srivastava, Vidu Garg

**Affiliations:** 1 Center for Cardiovascular and Pulmonary Research and the Heart Center, Nationwide Children's Hospital, The Ohio State University, Columbus, Ohio, United States of America; 2 Department of Pediatrics, University of Texas Southwestern Medical Center, Dallas, Texas, United States of America; 3 Department of Pediatrics, The Ohio State University, Columbus, Ohio, United States of America; 4 Department of Cardiology, Children's Hospital Boston and Harvard Medical School, Boston, Massachusetts, United States of America; 5 Gladstone Institute of Cardiovascular Disease, University of California San Francisco, San Francisco, California, United States of America; 6 Department of Pediatrics, University of California San Francisco, San Francisco, California, United States of America; 7 Department Biochemistry and Biophysics, University of California San Francisco, San Francisco, California, United States of America; 8 Department of Molecular Genetics, The Ohio State University, Columbus, Ohio, United States of America; Vanderbilt University, United States of America

## Abstract

Defects of atrial and ventricular septation are the most frequent form of congenital heart disease, accounting for almost 50% of all cases. We previously reported that a heterozygous G296S missense mutation of *GATA4* caused atrial and ventricular septal defects and pulmonary valve stenosis in humans. *GATA4* encodes a cardiac transcription factor, and when deleted in mice it results in cardiac bifida and lethality by embryonic day (E)9.5. *In vitro*, the mutant GATA4 protein has a reduced DNA binding affinity and transcriptional activity and abolishes a physical interaction with TBX5, a transcription factor critical for normal heart formation. To characterize the mutation *in vivo*, we generated mice harboring the same mutation, *Gata4 G295S*. Mice homozygous for the *Gata4 G295S* mutant allele have normal ventral body patterning and heart looping, but have a thin ventricular myocardium, single ventricular chamber, and lethality by E11.5. While heterozygous *Gata4 G295S* mutant mice are viable, a subset of these mice have semilunar valve stenosis and small defects of the atrial septum. Gene expression studies of homozygous mutant mice suggest the G295S protein can sufficiently activate downstream targets of Gata4 in the endoderm but not in the developing heart. Cardiomyocyte proliferation deficits and decreased cardiac expression of *CCND2*, a member of the cyclin family and a direct target of Gata4, were found in embryos both homozygous and heterozygous for the *Gata4 G295S* allele. To further define functions of the *Gata4 G295S* mutation *in vivo*, compound mutant mice were generated in which specific cell lineages harbored both the *Gata4 G295S* mutant and *Gata4* null alleles. Examination of these mice demonstrated that the Gata4 G295S protein has functional deficits in early myocardial development. In summary, the *Gata4 G295S* mutation functions as a hypomorph *in vivo* and leads to defects in cardiomyocyte proliferation during embryogenesis, which may contribute to the development of congenital heart defects in humans.

## Author Summary

Cardiac malformations occur due to abnormal heart development and are the most prevalent human birth defect. Defects of atrial and ventricular septation are the most common type of congenital heart defect and are the result of incomplete closure of the atrial and ventricular septa, a process required for formation of a four-chambered heart. The molecular mechanisms that underlie atrial and ventricular septal defects are unknown. We previously published a highly penetrant autosomal dominant mutation (G296S) in *GATA4*, which was associated with atrial and ventricular septal defects in a large kindred. The disease-causing mutation has a spectrum of biochemical deficits affecting both DNA binding and protein–protein interactions. Here, we report the generation and phenotypic characterization of mice harboring the orthologous mutation in *Gata4* (G295S). While homozygous mutant mice display embryonic lethality and cardiac defects, the phenotype is less severe than *Gata4-null* mice. A subset of *Gata4 G295S* heterozygote mice display a persistent interatrial communication (patent foramen ovale) and stenosis of the semilunar valves. Molecular characterization of the mutant mice suggests that the Gata4 G295S mutant protein results in diminished expression of Gata4 target genes in the heart and functional deficits in cardiomyocyte proliferation. Thus, cardiomyocyte proliferation defects may contribute to defects of cardiac septation found in humans with *GATA4* mutations.

## Introduction

Congenital heart defects (CHD) are the most prevalent of all human birth defects with an estimated incidence of 6–8 per 1,000 live births [1], . Defects of cardiac septation, which encompass atrial and ventricular septal defects, may occur as an isolated defect or in combination with other cardiac malformations. Defects of atrial and ventricular septation are the most common type of CHD and account for 50% of all cases of CHD. If unrepaired, these defects result in ventricular dilation and heart failure, pulmonary overcirculation leading to pulmonary vascular disease, atrial enlargement predisposing to atrial arrhythmias and ultimately a decreased life expectancy. The etiology for atrial and ventricular septal defects is multifactorial with genetic and environmental factors playing important roles [3], [4].

Monogenic etiologies for atrial and ventricular septal defects have been primarily discovered by studying large families with autosomal dominant forms of septal defects using traditional linkage approaches [5], [6]. The first genetic etiology for atrial septal defects was the disovery that mutations in the transcription factor, TBX5, are a cause of septation defects in the setting of Holt-Oram syndrome, which is characterized by cardiac and upper limb malformations [7]. *Tbx5* haploinsufficiency in mice accurately mimics the phenotype found in patients with Holt-Oram syndrome [8]. Mutations in the cardiac transcription factor, NKX2-5, were identified in families who primarily exhibited non-syndromic atrial septal defects and atrioventricular conduction abnormalities [9]. While targeted deletion of *Nkx2-5* in mice causes developmental arrest during heart tube looping, haploinsufficiency of *Nkx2-5* results in only subtle defects of atrial septation [10], [11]. Similarly, mutations in the cardiac transcription factor, GATA4, have also been linked to atrial and ventricular septal defects [12], [13], [14], [15], [16]. Gata4 is necessary for normal cardiac development as mice with targeted deletion of *Gata4* display embryonic lethality and defects in ventral morphogenesis associated with failure to form a single ventral heart tube [17], [18]. Subsequent studies have demonstrated that Tbx5, Nkx2-5, and Gata4 interact to regulate distinct developmental processes during heart development [19], [20], [21]. While many of the human mutations are predicted to result in haploinsufficiency, little is understood about the underlying mechanism by which reduced transcription factor dosage causes defects in cardiac septation.

We reported a large pedigree with autosomal dominant congenital heart disease that was associated with a mutation of a highly conserved glycine residue to a serine at codon 296 (G296S) [12]. The affected family members had a spectrum of cardiac phenotypes, primarily atrial and ventricular septal defects and pulmonary valve stenosis [12]. *In vitro* experiments demonstrated that the mutant Gata4 protein had a greatly reduced affinity for its binding element with an associated decrease in transcriptional activity and disrupted a novel interaction between Gata4 and Tbx5 [12]. Subsequently, two other families have been reported with a *GATA4 G296S* mutation, and affected members display a similar phenotype of atrial septal defects and incompletely penetrant pulmonary valve stenosis [22]. While Gata4 has been shown to be important for several critical processes during heart development [23], [24], [25], [26], the pathogenesis of heart malformations in humans with the G296S missense mutation in *GATA4* is not as well understood.

To identify the functional deficits of the *GATA4 G296S* mutation *in vivo*, we generated and characterized transgenic mice that contain the orthologous G295S mutation in the *Gata4* murine locus. Here, we show that homozygous *Gata4 G295S* knock-in (ki) mice (*Gata4 G295S^ki/ki^*) display normal ventral morphogenesis but early embryonic lethality after the linear heart tube stage. Histologic analysis demonstrates a thin ventricular myocardium in the *Gata4 G295S^ki/ki^* embryos, which is associated with a cardiomyocyte proliferation defect. Molecular characterization of these mutant embryos demonstrates that expression of Gata4 target genes is decreased in the heart. Echocardiographic examination of heterozygote (*Gata4 G295S^ki/wt^*) mice found subtle atrial septal defects and semilunar valve stenosis. Embryonic cardiomyocytes from heterozygote mice display cardiac proliferation deficits. Consistent with functional deficits for the *Gata4 G295S* mutation, compound heterozygote mice harboring only a single *Gata4 G295S* mutant allele in the early myocardium recapitulated the phenotype seen in *Gata4 G295S^ki/ki^* embryos. These studies demonstrate the generation of a mouse model for human cardiac malformations and suggest that abnormal cardiomyocyte proliferation may contribute to human atrial and ventricular septal defects caused by mutations in *GATA4*.

## Results

### Generation and phenotypic characterization of heterozygote *Gata4 G295S^ki/wt^* mice

**Figure 1:**
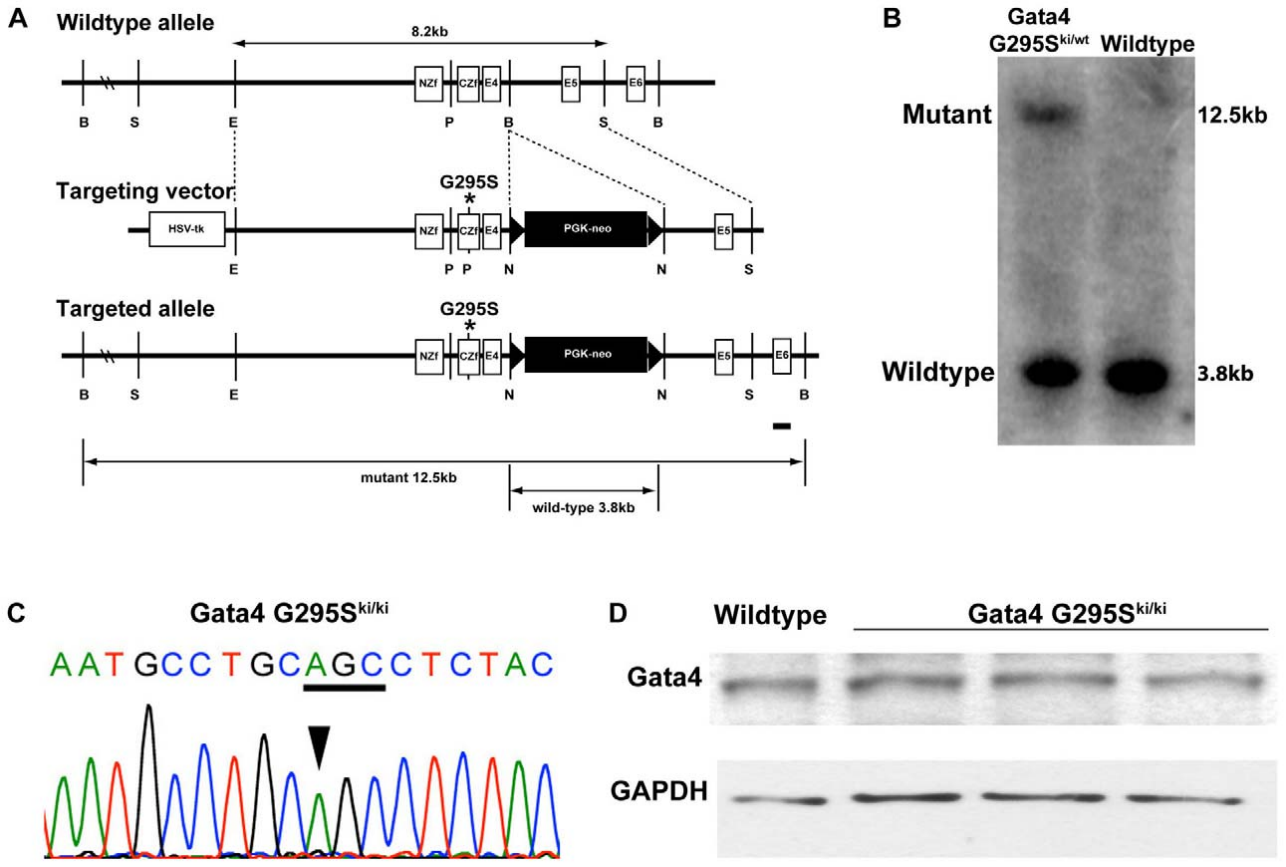
Targeting strategy for generation of *Gata4 G295S* knock-in mice. (A) Single nucleotide change resulting in the glycine to serine mutation was introduced into the mouse *Gata4* locus. Partial restriction map of the murine *Gata4* wildtype allele (top), the *Gata4* targeting vector (middle), and successfully targeted allele (bottom) are shown. Homologous recombination results in replacement of wildtype *Gata4* with genomic DNA harboring a substitution of glycine to serine at position 295 into the mouse *Gata4* locus, as well as the incorporation of neomycin cassette surrounded by loxP sites. *Gata4* coding exons are shown as empty boxes, whereas the exon used as a probe used for Southern blot analysis is highlighted by a black bar. NZf, amino- terminal zinc finger (exon 2); CZf, carboxy- terminal zinc finger (exon 3); E4, exon 4; E5, exon 5; E6, exon 6; B, BglI; S, SacI; E, EcoRV; and N, NotI. (B) Germline transmission of mutant allele was confirmed by Southern blotting after digestion of genomic DNA from *Gata4 G295S^ki/wt^* and wiltype mice with BglI. A 3.8 kb wildtype band and a 12.5 kb mutant band using 3′ external probe are shown (black bar in A). (C) Direct sequencing confirmed the presence of mutated residue that altered glycine (GGC) to serine (AGC) in DNA from *Gata4 G295S^ki/ki^* embryos. (D) Western blotting demonstrates that levels of Gata4 protein are equivalent in *Gata4 G295S^ki/ki^* hearts (from three different embryos) as compared to wildtype E9.5 hearts. Equal protein loading is shown by Western blotting to GAPDH.

In order to determine the *in vivo* functional deficits of the human CHD-causing *GATA4 G296S* mutation, we generated mice harboring the orthologus mutation at codon 295 in the murine *Gata4* gene (G295S). Using a previously published targeting construct, we mutated the nucleotide from G to A resulting in a glycine to serine substitution in exon 3 which contains the C-terminal zinc finger of Gata4 [27] (Figure 1A). Successfully targeted ES cell clones were identified by Southern analysis and direct sequencing and injected into host blastocysts to generate chimeras. Germline transmission of the targeted G295S knock-in allele (G295S^ki^) was detected by Southern blotting in chimeric mice and confirmed by direct sequencing in heterozygous and homozygous mice (Figure 1B–1C and data not shown).

**Figure 2:**
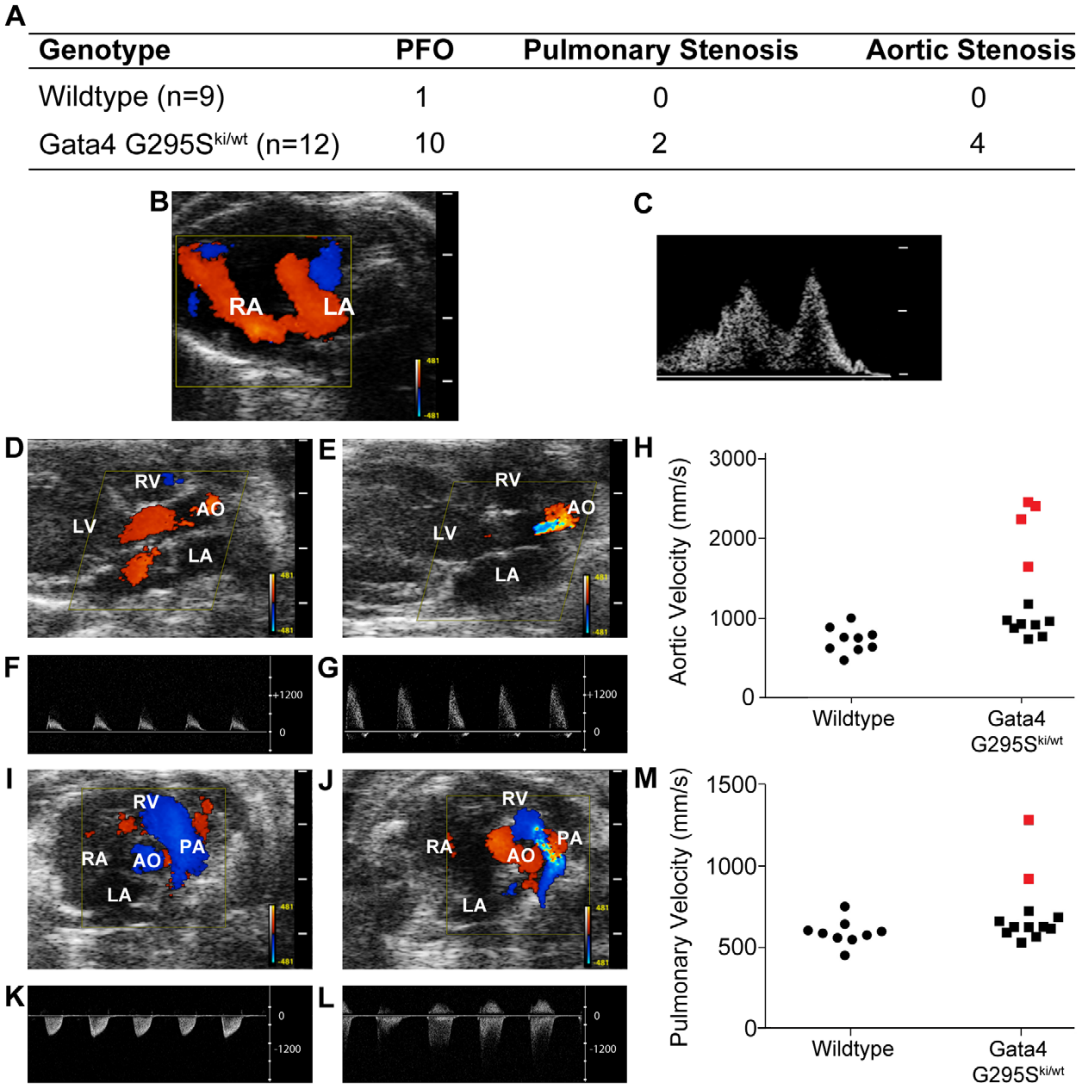
Atrial septal defects and semilunar valve stenosis in *Gata4 G295S^ki/wt^* mice. (A) Table showing the frequency of cardiac abnormalities identified in *Gata4 G295S^ki/wt^* mice (n = 9) and wildtype littermates (n = 12). Representative images of color (B, D, E, I, J) and pulsed wave Doppler (C, F, G, K, L) findings are shown. Small atrial communication demonstrated by both (B) color and (C) pulsed wave Doppler in *Gata4 G295S^ki/wt^* mouse. Color Doppler recordings across normal aortic valve of a wildtype mouse (D) and stenotic aortic valve of *Gata4 G295S^ki/wt^* mouse (E). Pulsed Doppler waveforms of flow across the aortic valve in wildtype (F) and *Gata4 G295S^ki/wt^* (G) mice demonstrate increased aortic velocity in mutant mice. (H) Scatter plot showing aortic velocities in wildtype and *Gata4 G295S^ki/wt^* mice. Four *Gata4 G295S^ki/wt^* mice with aortic stenosis are indicated in red. Color Doppler recordings across pulmonary valve of a wildtype (I) and stenotic pulmonary valve in *Gata4 G295S^ki/wt^* mice (J). Pulsed Doppler waveforms across pulmonary valve of wildtype (K) and *Gata4 G295S^ki/wt^* mice (L) show increased velocity in mutant mice. (M) Scatter plot showing velocity across pulmonary valve in wildtype and *Gata4 G295S^ki/wt^* mice. Two *Gata4 G295S^ki/wt^* mice with pulmonary stenosis indicated in red.

Although the *Gata4 G295S^ki/wt^* heterozygote mice appeared grossly normal, we examined these mutant mice for cardiac structural and functional abnormalities using transthoracic echocardiography. In a genotype-blinded fashion, M-Mode, 2-D pulsed and color-flow Doppler studies were performed in 8 and 16 week old *Gata4 G295S^ki/wt^* mice and their wildtype littermates. Intermittent shunting of blood was noted between the left and right atria in 10/12 *Gata4 G295S^ki/wt^* mice as compared to only 1/9 wildtype littermates (p value<0.05) (Figure 2A–2C). The intermittent atrial communication defect is a patent foramen ovale, which was also found in mice heterozygous for *Nkx2-5*, a gene also implicated in human atrial septal defects [11]. Quantitative pulsed Doppler recordings across the pulmonary and aortic valves demonstrated mild aortic stenosis in 4/12 *Gata4 G295S^ki/wt^* mice and pulmonary stenosis in 2/12 *Gata4 G295S^ki/wt^* mouse (Figure 2A and 2D–2M). No evidence of aortic or pulmonary valve stenosis was noted in wildtype littermates (p value<0.05). The left ventricular function, chamber size and wall thickness in *Gata4 G295S^ki/wt^* mice was not statistically different from wildtype littermates (Figure S1). Histologic analysis of *Gata4 G295S^ki/wt^* hearts demonstrated the patent foramen ovale (Figure S2A–S2D) along with thickened aortic (Figure S2E–S2F) and pulmonary valve leaflets (Figure S2G–S2H).

### Phenotypic characterization of homozygous *Gata4 G295S^ki/ki^* mice

To determine the *in vivo* functional deficits of the *Gata4 G295S* mutation, we interbred *Gata4 G295S^ki/wt^* to generate *Gata4 G295S^ki/ki^* mice. Analysis at postnatal day 7 demonstrated no *G295S^ki/ki^* pups indicating that the homozygous knock-in allele is embryonic or early neonatal lethal. To determine the timing of lethality, we performed timed mating and found that *Gata4 G295S^ki/ki^* embryos did not survive beyond embryonic day (E)11.5, and normal Mendelian ratios were noted from E8.5–E10.5 (Table 1). To determine the expression levels of Gata4 mutant protein in *Gata4 G295S^ki/ki^* embryos, we extracted protein from the hearts of E9.5 *Gata4 G295S^ki/ki^* embryos and wildtype littermates. Immunoblotting demonstrated that total Gata4 protein levels in *G295S^ki/ki^* embryos were unchanged as compared to wildtype littermates, suggesting that the G295S mutant mRNA was not undergoing decay and resulting in a *Gata4-*deficient mouse (Figure 1D) [28].

**Table 1:** Distribution of progeny obtained from intercrossing Gata4 G295S heterozygote mice.

	Wildtype	*Gata4 G295S^ki/wt^*	*Gata4 G295S^ki/ki^*
P7	21 (33%)	43 (67%)	0 (0%)
E12.5–E14.5	10 (45%)	12 (55%)	0 (0%)
E11.5	9 (20%)	31 (71%)	4 (9%)
E10.5	16 (32%)	24 (47%)	11 (21%)
E9.5	62 (25%)	134 (54%)	52 (21%)
E8.5–E9.0	6 (22%)	15 (56%)	6 (22%)

**Figure 3:**
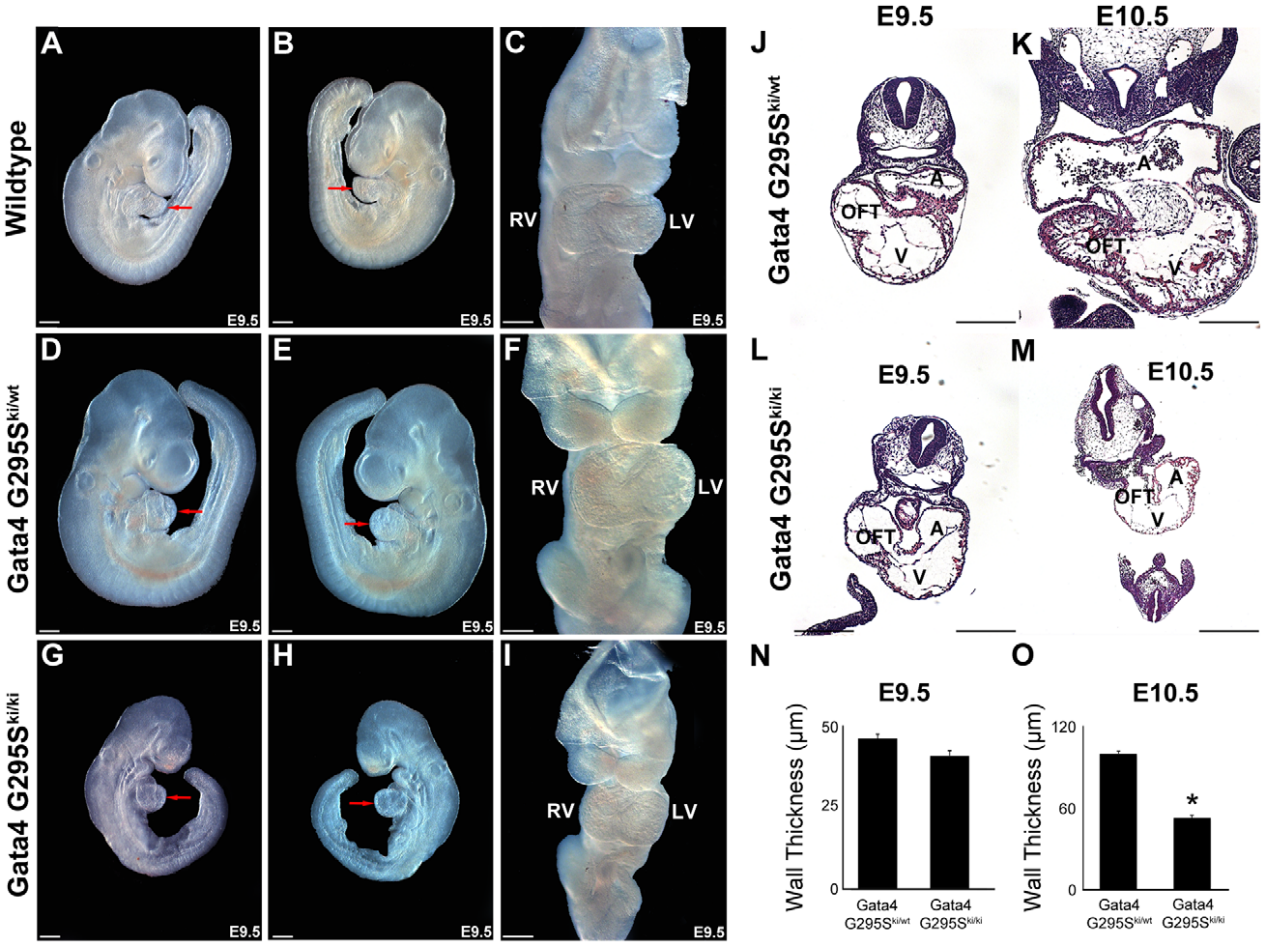
*Gata4 G295S^ki/ki^* mice display growth retardation and a thin myocardium. Right (A,D,G), left (B,E,H) and frontal (C,F,I) views of embryos are shown. Growth retardation of *Gata4 G295S^ki/ki^* E9.5 embryos (G,H) when compared to wildtype (A,B) and heterozygote littermates (D,E). A fused heart tube with proper looping is found in *Gata4 G295S^ki/ki^* embryos (I) simlar to wildtype (C) and *Gata4 G295S^ki/wt^* (F) littermates. Coronal sections through *Gata4 G295S^ki/wt^* (J,K) and *Gata4 G295S^ki/ki^* (L,M) embryos at E9.5 (J,L) and E10.5 (K,M). Normal myocardial thickness is found at E9.5 in homozygous mutant embryos (L) while thin myocardium is seen at E10.5 (M) when compared to heterozygote littermate (K). Quantification of ventricular wall thickness in *Gata4 G295S^ki/wt^* and *Gata4 G295S^ki/ki^* embryos at E9.5 (N) and 10.5 (O). RV, right ventricle; LV, left ventricle; red arrow, heart; A, atria; V, ventricle; OFT, outflow tract. Scale bars indicate 200 µm.

Gross examination of *Gata4 G295S^ki/ki^* embryos demonstrated severe growth retardation compared to wildtype and heterozygote littermates at E9.5 and E10.5 (Figure 3 and Figure S3). The mutant embryos did not display defective heart tube fusion or cardiac bifida, as described in mice with targeted deletion of *Gata4* [17], [18]. The mutant embryos displayed a linear heart tube and variable amounts of cardiac looping, with some appearing normal while others demonstrate incomplete or delayed looping (Figure 3A–3I and Figure S3).

To further define the morphologic defects in *Gata4 G295S^ki/ki^* embryos, histologic examination of wildtype and mutant embryos was performed. The embryonic hearts of knock-in homozygotes did not reveal any obvious defects in cardiac morphogenesis at E9.0 and E9.5 (Figure 3J, 3L and 3N and data not shown). However by E10.5, severe thinning of myocardium and associated decreased wall thickness was noted in the ventricles of *Gata4 G295S^ki/ki^* embryos compared with heterozygote littermate controls (Figure 3K, 3M and 3O). The delayed lethality and the presence of a fused heart tube as compared to *Gata4-null* mice suggested that the Gata4 G295S mutant protein was functioning as a hypomorph *in vivo*.

### Expression of Gata4 target genes

**Figure 4:**
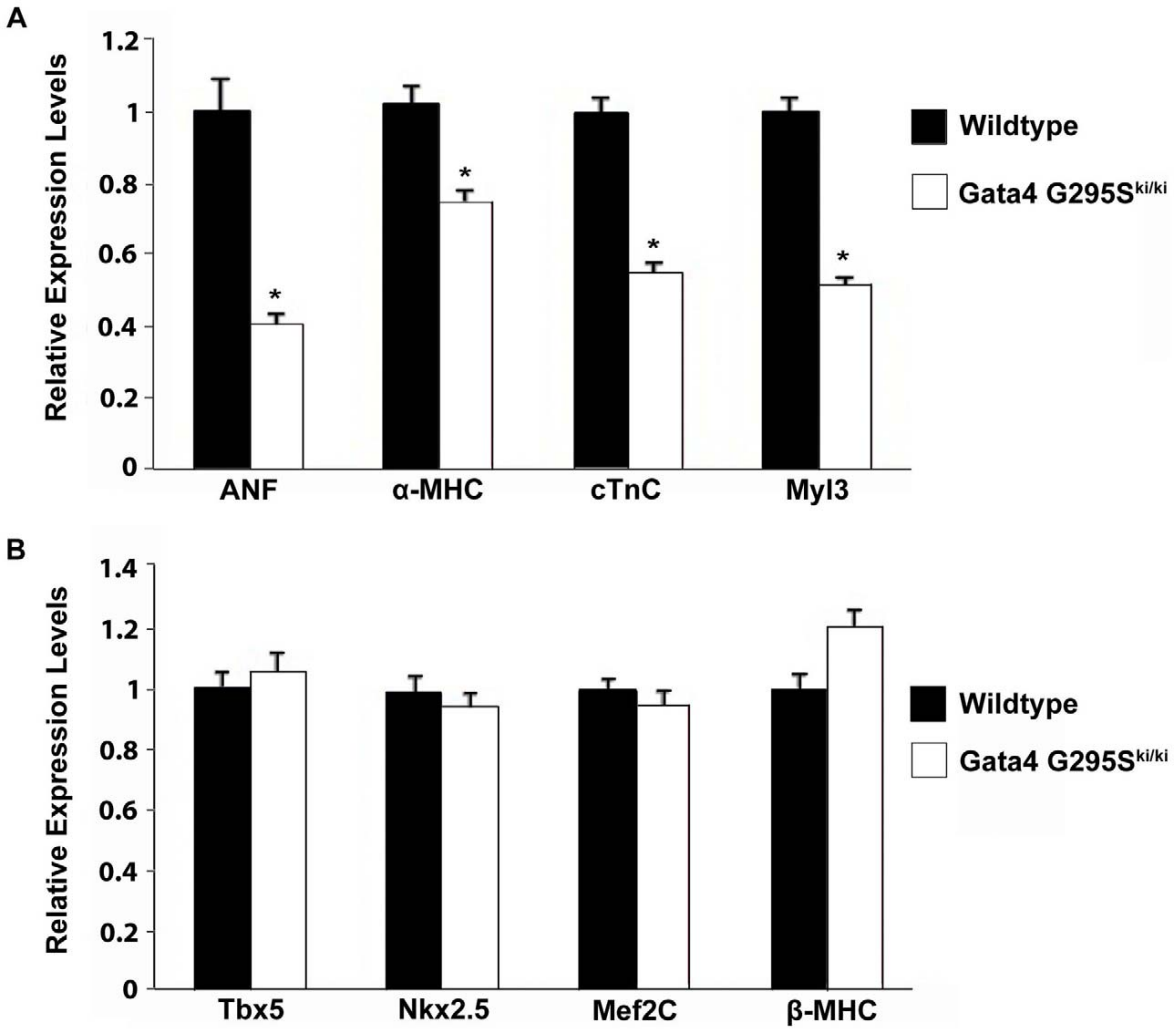
Decreased expression of Gata4 target genes in *Gata4 G295S^ki/ki^* embryonic hearts. (A) Downregulation of ANF, α-MHC, cTnC, and Myl3 in homozygous mutant E9.5 hearts as compared to wildtype littermates as measured by qRT-PCR. *, p value<0.05. (B) Quantitative RT-PCR demonstrates no significant change in expression levels of Tbx5, Nkx2.5, Mef2C and β-MHC in E9.5 *Gata4 G295S^ki/ki^* hearts when compared to wildtype littermates.

In the early embryo, Gata4 is expressed in developing heart along with the visceral and parietal endoderm [29]. Numerous cardiac genes, including α-myosin heavy chain (α-MHC), cardiac troponin-C (cTNC), atrial natriuretic factor (ANF), have been shown to be direct transcriptional targets of Gata4 [29]. *In vitro* transactivation studies suggested that the Gata4 G295S mutation had decreased ability to activate downstream target genes [12]. To determine if the expression of direct transcriptional targets of Gata4 were altered in the *Gata4 G295S^ki/ki^* mutant hearts, we extracted RNA from E9.5 hearts and analyzed the expression of α-MHC, cTnC, ANF, and myosin light chain 3 (Myl3) by quantitative RT-PCR. The expression levels of all four genes were significantly decreased in *Gata4 G295S^ki/ki^* mutant hearts as compared to wildtype controls (Figure 4A). In contrast, the expression of non-Gata4 target genes, Tbx5 and β-MHC, was unchanged (Figure 4B). Of note, the expression of Nkx2.5, and Mef2c, cardiac transcription factors which are proposed to be direct Gata4 targets, was also unchanged (Figure 4B) [30], [31]. We also did not find any change in expression of Gata5 and Gata6 by quantitative RT-PCR (Figure S4). Radioactive *in situ hybridization* was performed to determine if cardiomyoctyes in *Gata4 G295S^ki/ki^* hearts displayed normal markers of differentiation. At E9.5, the expression of *Tbx5*, *Hand1*, and *Hand2* was similar in *G295S^ki/ki^* hearts as compared to control littermates (Figure S5).

The cardiac bifida found in *Gata4-null* embryos occurs secondary to loss of Gata4 in the embryonic endoderm [17], [18], [32], [33], [34]. *Gata4 G295S^ki/ki^* embryos undergo normal heart tube fusion, suggesting that they overcome this endoderm-mediated defect. We examined the expression of Gata4-responsive endoderm genes, alpha-fetoprotein (*Afp*) and *Sox17*, along with the endoderm transcription factors, *Hex1* and *Hnf4*, that are not direct transcriptional targets of *Gata4* [35], [36]. *Afp* and *Sox17* did not have decreased levels of expression by qRT-PCR in *Gata4 G295S^ki/ki^* embryos when compared to wildtype littermates at three different embryonic timepoints (Figure S6). Actually, the expression levels of Afp and Sox17 were somewhat increased in *G295S^ki/ki^* embryos similar to Hex1 and Hnf4, but this maybe secondary to the growth retardation found in mutant embryos (Figure S6).

### Gata4 G295S mutation and deficits in cardiomyocyte proliferation

**Figure 5:**
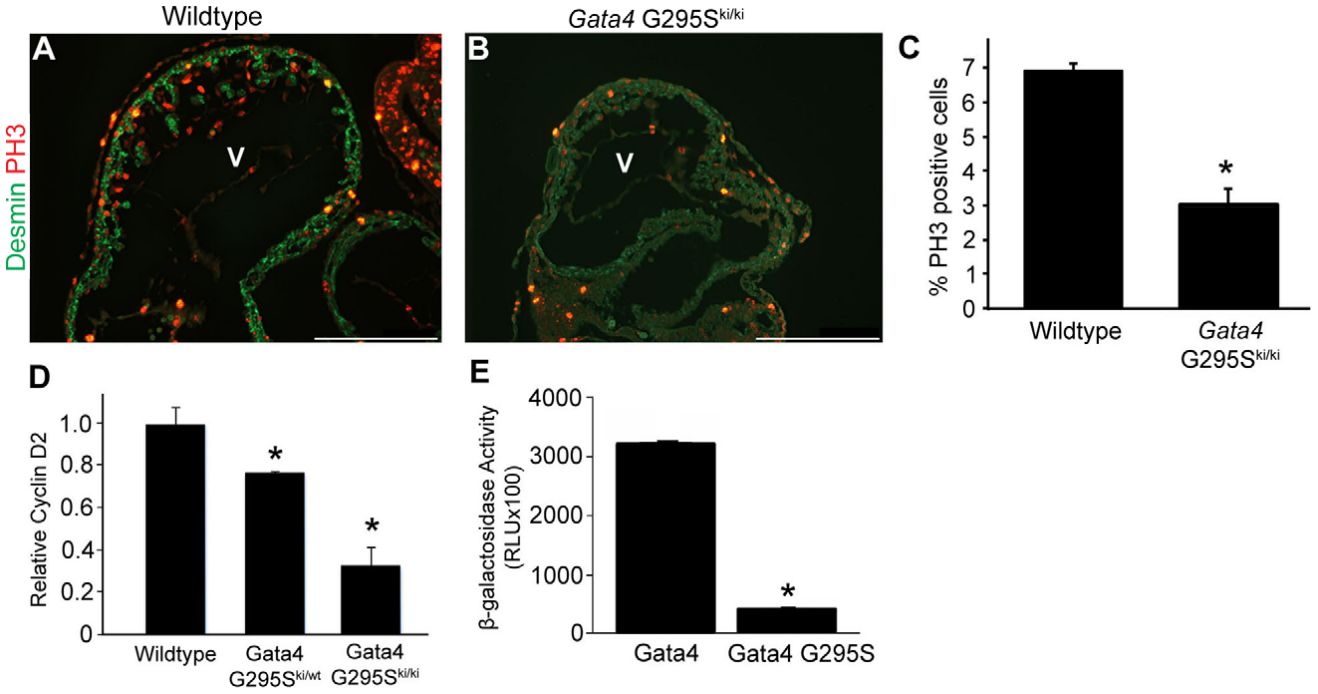
G295S mutation in *Gata4* results in cardiomyocyte proliferation defects. Immunofluorescent staining for phosphohistone H3 (red) along with desmin staining for cardiomyocytes (green) demonstrates decreased cardiomyocyte proliferation in *Gata4 G295S^ki/ki^* (B) as compared to wildtype (A) in histologic sections of E9.5 hearts. V, ventricle. (C) Quantification of phosphohistone H3 (PH3) staining of cardiomyocytes is decreased in *Gata4 G295S^ki/ki^* embryonic hearts when compared to wildtype. *, p value<0.05. (D) Quantitative RT-PCR demonstrates decreased cyclin D2 expression in the E9.5 hearts of both *Gata4 G295S^ki/wt^* and *Gata4 G295S^ki/ki^* embryos.*, p value<0.05. (E) Cyclin D2 β-gal reporter assays using wildtype Gata4 or Gata4 G295S mutant plasmids demonstrates that the G295S mutant protein has decreased transactivation ability as compared to wildtype Gata4. Error bars represent the standard deviation at least three independent experiments each performed in triplicate. *, p value<0.05. Scale bars indicate 200 µm.

In order to determine the etiology of the thin ventricular myocardium found in E10.5 *Gata4 G295S^ki/ki^* hearts, we measured the levels of cardiomyocyte proliferation and apoptosis in the mutant hearts. In *Gata4 G295S^ki/ki^* embryos, we observed decreased cardiomyocyte proliferation, as assessed by phosphohistone H3 staining at E9.5 in *Gata4 G295S^ki/ki^* embryos as compared to littermate controls (Figure 5A–5C), but no differences in apoptosis as assessed by TUNEL staining were found (data not shown). Consistent with this finding, the mRNA levels of cyclin D2, a direct transcriptional target of Gata4 that is critical for cell proliferation, was downregulated in homozygous knock-in embryos by qRT-PCR (Figure 5D) [37]. Additionally, the ability of the Gata4 G295S mutant protein to activate cyclin D2 beta-gal reporter in HeLa cells was significantly reduced as compared to wildtype Gata4 (Figure 5E).

**Figure 6:**
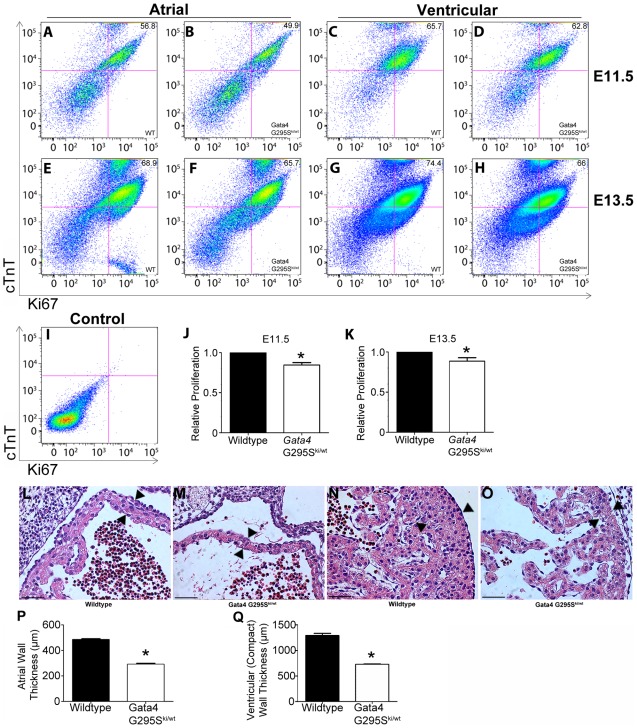
Proliferation deficits in *Gata4 G295S^ki/wt^* embryonic cardiomyocytes. (A–I) Cells were isolated from wildtype (WT) and *Gata4 G295S^ki/wt^* E11.5 and E13.5 hearts. FACS analyses for cardiac troponin T (cTnT)-positive cells was performed for (A, B) E11.5 atria, (C, D) E11.5 ventricles, (E, F) E13.5 atria, and (G,H) E13.5 ventricles. Proliferating cells are detected by staining with Ki67. Representative data are shown in each panel. (I) FACS analysis of unstained cells used as a control. Quantification of proliferative cardiomyocytes in *Gata4 G295S^ki/wt^* mutant hearts as compared to wildtype littermate hearts at E11.5 (J) and E13.5 (K). Experiments were performed in triplicate using pooled hearts and all data are presented as means ± standard deviation; *p value<0.05. Coronal sections through the heart of wildtype (L, N) and *Gata4 G295S^ki/wt^* (M, O) E12.5 hearts. High magnification images of the atria (L,M) and ventricle (N, O) are shown. Quantitative analysis demonstrates decreased wall thickness in the (P) atria and (Q) compact ventricular myocardium in *Gata4 G295S^ki/wt^* as compared to wildtype littermates (n = 3 for each genotype). Arrowheads, representative site of measurement; *, *p* value<0.05. Scale bars indicate 200 µm.

Interestingly, we found that mRNA levels of cyclin D2 were also decreased in the hearts of heterozygote *Gata4 G295S^ki/wt^* embryos (Figure 5D). In order to determine if subtle cardiomyocyte proliferation deficits existed in *Gata4 G295S^ki/wt^* embryos, we assessed cell proliferation utilizing a fluorescence activated cell sorting (FACS)-based strategy. Hearts were dissected from E11.5 and E13.5 embryos and cardiomyocytes were purified based on cardiac troponin-T expression [38]. *Gata4 G295S^ki/wt^* embryonic atrial and ventricular cardiomyocytes displayed decreased cell proliferation as compared to cardiomyocytes from wildtype littermates at both E11.5 and E13.5 (Figure 6A–6K). Accordingly, we found that *Gata4 G295S^ki/wt^* embryos had thinner atrial and compact ventricular layers at E12.5 (Figure 6L–6Q).

### *In vivo* analysis of cell lineage deficits of the Gata4 G295S protein

**Figure 7:**
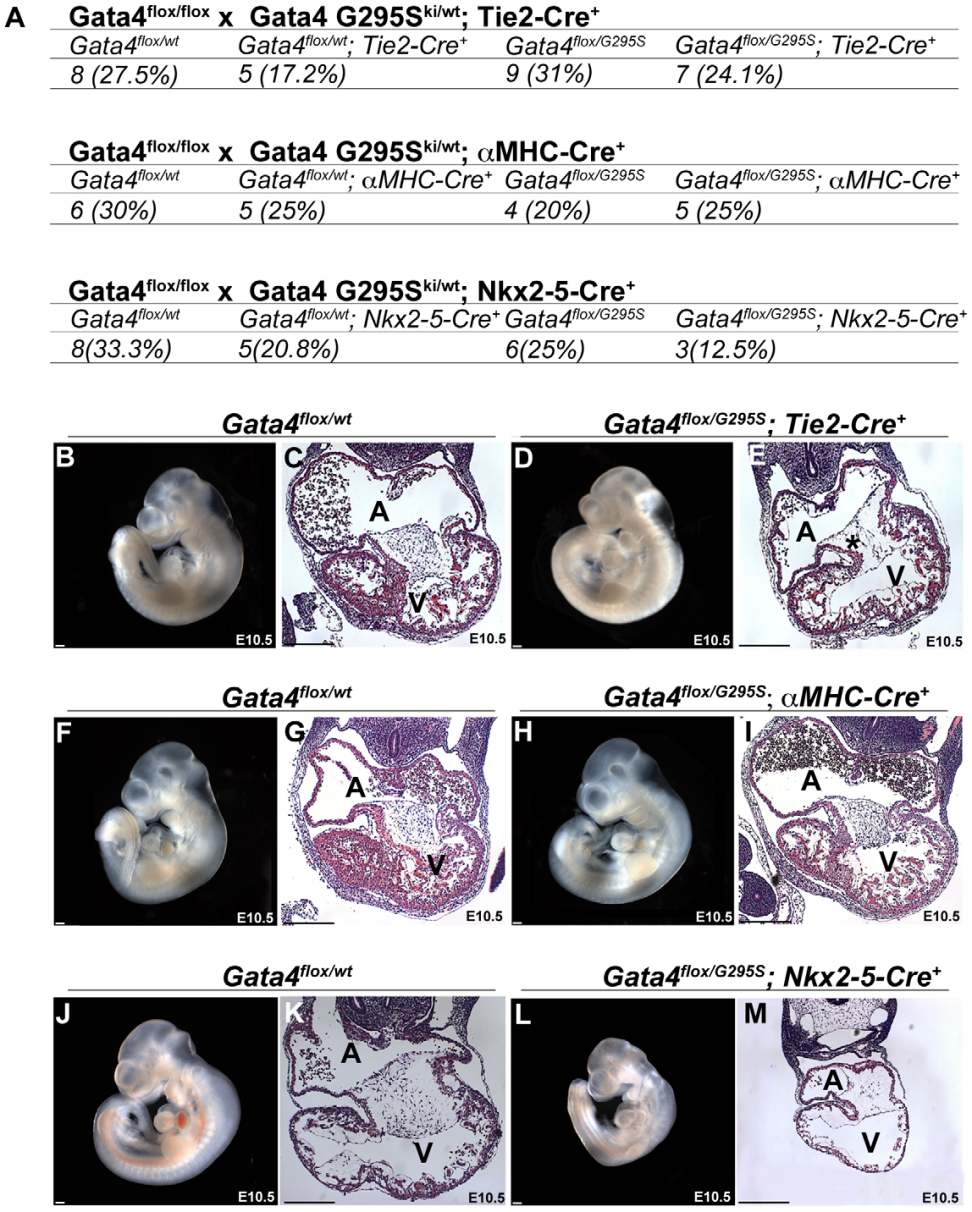
*Gata4 G295S* mutation has *in vivo* functional deficits in the early embryonic myocardium. (A) Embryonic lethality by E10.5 was found in compound heterozygote mice expressing only a *Gata4 G295S* mutant allele in early myocardium with the *Nkx2-5-Cre*, but normal Mendelian ratios were seen when the *Gata4 G295S* mutant allele was expressed in endocardium and late myocardium using *Tie2-Cre* and α-*MHC-Cre*, respectively. Images (B, D, F, H, J, L) and histologic sections (C, E, G, I, K, M) of E10.5 embryos generated with *Tie2-Cre*, which is specific for endocardium (B–E); α*-MHC-Cre*, which is specific for late embryonic myocardium (F–I); and *Nkx2.5-Cre*, which is specific for early embryonic myocardium (J–M), are shown. (L,M) Growth retardation and myocardial thinning were seen in *Gata4 G295S^ki/flox^*; *Nkx2-5-Cre^+^* E10.5 embryos similar to the phenotype of the *Gata4 G295S^ki/ki^* embryo. (H,I) The hearts of *Gata4 G295S^ki/flox^*; α*-MHC-Cre^+^* appeared normal at E10.5. (D,E) While the *Gata4 G295S^ki/flox^*; *Tie2-Cre^+^* did not show growth retardation or myocardial thinning, hypocellular endocardial cushions were noted (*). A, atria; V, ventricle; scale bars indicate 200 µm.

To assess the functional deficit of the Gata4 G295S mutant protein in different cell lineages *in vivo*, we first generated mice that were compound heterozygotes for the *Gata4 G295S* mutant allele and a tissue-specific *Cre*, and lineage-specific deletion of the *Gata4* was performed by crossing them with mice with a floxed *Gata4* allele [23]. One fourth of the resultant progeny were predicted to harbor only the mutant allele in specific cardiac cell types. Tissue specific deletion was obtained by expressing *Cre* under the regulation of Tie2, which is expressed in endocardium, endothelium along with a subset of hematopoietic cells (presumed to be circulating endothelial progenitor cells); αMHC, which is specific for late embryonic myocardium, with robust Cre-mediated excision starting at E9.5; and Nkx2-5, which is expressed in early embryonic myocardium starting at E8.0 and also in the pharynx and liver [39], [40], [41]. Immunohistochemistry for Gata4 demonstrated decreased Gata4 expression in E10.5 embryonic hearts with the Nkx2-5 Cre (Figure S7A–S7B) and Tie2-Cre (Figure S7C–S7D) in the myocardium and endocardium, respectively. With the αMHC-Cre, expression of Gata4 was only mildly decreased in the myocardium likely related to the later onset of Cre expression (Figure S7E–S7F). Analysis of these compound heterozygotes allowed for the identification of functional deficits of the Gata4 G295S protein that were present specific to the early myocardium (emGata4G295S), late myocardium (lmGata4G295S) and endocardium (enGata4G295S). We found that only the emGata4G295S was lethal by post-natal day 10 while the lmGata4G295S and enGata4G295S demonstrated partial lethality (Table S1). Genotyping of E10.5 embryos from the crosses using the Tie2-*Cre* (enGata4G295S) and αMHC-*Cre* (lmGata4G295S) showed the expected genotypes were present in normal Mendelian ratios with no evidence of growth retardation (Figure 7A–7I). This contrasts with the severe growth retardation found in *Gata4G295S^ki/ki^* embryos (Figure 3 and Figure S3). However, embryos obtained from crosses with *Gata4^flox/flox^* and *Gata4 G295S^ki/wt^*;Nkx2-5-*Cre^+^* (emGata4G295S) demonstrated partial lethality by E10.5 and evidence of severe growth retardation (Figure 7A and 7J–7M). Histologic examination of these three genotypes demonstrated a normal myocardial thickness in enGata4G295S and lmGata4G295S, but myocardial thinning in emGata4G295S embryos (Figure 7E, 7I, and 7M), similar to *Gata4 G295S^ki/ki^* embryos (Figure 3K and 3M). These studies support that the Gata4 G295S mutant protein has functional deficits in the early myocardium that contributes to a thin myocardium.

## Discussion

The *GATA4 G296S* mutation has been associated with atrial septal defects and pulmonary valve stenosis in multiple human families [12], [22]. *In vitro* studies suggested that GATA4 G296S mutant protein resulted in specific functional deficits including diminished DNA binding affinity, reduced transcriptional activity and loss of a protein-protein interaction with TBX5. Here, we have generated a knock-in mouse harboring the corresponding G295S mutation in *Gata4*. Phenotypic characterization of these mice demonstrate that the Gata4 G295S mutation functions as a hypomorph *in vivo* as evidenced by the *Gata4 G295S^ki/ki^* embryos displaying prolonged survival as compared to *Gata4-null* embryos and decreased expression of Gata4 transcriptional target genes. Consistent with the cardiac phenotype seen in humans with *GATA4* mutations, *Gata4 G295S^ki/wt^* mice also display cardiac abnormalities. In addition, we found that the G295S mutation of *Gata4* results in defects of embryonic cardiomyocyte proliferation both *in vitro* and *in vivo*. These findings suggest a potential role for abnormal cardiomyocyte proliferation in the development of atrial and ventricular septal defects caused by mutations in *GATA4*.

The G295S mutation in Gata4 may result in multiple functional deficits in the embryonic heart. Mice homozygous for the *Gata4 G295S* mutation suffer embryonic lethality before E10.5, which limited our analysis to early myocardial development and precluded analysis of potential functional deficits at later stages of heart development. Conditional deletion of *Gata4* in the endocardium results in defective endocardial-mesenchymal transition (EMT) and hypoplastic endocardial cushions [26]. In addition, we have previously shown the G295S mutation in *Gata4* inhibits an interaction with Tbx5 and *Gata4-Tbx5* compound heterozygotes display atrioventricular septal defects [12], [21]. It remains unclear if the *GATA4 G296S* mutation has a role in formation of the endocardial cushions but one family member with the G296S mutation did have an atrioventricular septal defect [12] and other non-related individuals with *GATA4* mutations have atrioventricular septal defects [13]. In our analysis of potential cell lineage deficits for the Gata4 G295S protein, we did find that enGata4G295S mice, where the endocardium/endothelium expresses only the mutant protein, have somewhat hypocellular endocardial cushions and display partial lethality by post-natal day 10. This finding suggests that the Gata4 G295S mutation may have functional deficits in the endocardium, potentially by disrupting EMT but additional study is required (Figure 7). A role for Gata4 and Tbx5 in the endocardium for formation of the atrial septum by regulation of endothelial nitric oxide synthase (*Nos3*) has been proposed, but the early lethality of the *Gata4 G295S^ki/ki^* embryos did not allow for analysis of this pathway [42]. While this early lethality precluded examination of the development of the cardiac outflow tract and semilunar valves in the *Gata4 G295S^ki/ki^* embryos, the *Gata4 G295S^ki/wt^* heterozygotes did develop stenosis of the semilunar valves. This phenotype along with recent publications demonstrating bicuspid aortic valve and aortic valve stenosis in Gata5-null and Gata4;Gata5 compound heterozygote mice suggest that endocardial specific deficits of the G296S protein may contribute to the aortic and pulmonary valve stenosis [43], [44].

The phenotypic analysis of *Gata4 G295S^ki/ki^* mice demonstrates that the Gata4 G295 mutant allele is not a loss of function allele. Initial human genetic studies demonstrated that haploinsufficiency of *GATA4* resulted in cardiac malformations and there was a possibility that the *Gata4 G295* was a null allele [45]. Our data demonstrate that the G295S mutation in *Gata4* results in a selective loss of some Gata4 functions. Specifically, the Gata4 G295S protein is able to activate downstream target genes in the developing endoderm but not in the cardiac mesoderm. Potential mechanisms for this difference may lie in the inability of Gata4 G295S protein to interact with Tbx5 in the mesoderm to activate downstream targets or that Gata4 may function as a transcriptional co-activator in the endoderm and DNA binding is not necessary. The findings from the conditional deletion crosses, as discussed above, also support that the G295S mutant protein has defined functional deficits in different cell lineages. Additional investigation is needed to identify all the potential functions of Gata4 and then determine which are loss with the Gata4 G295S mutation.

Cardiomyocyte proliferation is critical for normal cardiac development, and our findings provide evidence that the *Gata4 G295S* mutation results in myocardial hypoplasia due to diminished cardiac proliferation. This phenotype is at least partially mediated by reduced expression of cyclin D2, a member of the D-cyclin family of cell cycle regulators. Mice lacking any single D-cyclin are viable and do not display obvious cardiac defects [46]. However, compound mutation of all three D-cyclin genes results in embryonic lethality due to cellular proliferation defects, including reduced cardiomyocyte cell division [47]. Our data demonstrate that both homozygote and heterozygote *Gata4 G295S^ki^* mice display cardiomyocyte proliferation deficits and suggest that this is a possible mechanism for the atrial septal defects seen in these mice.

The generation of the *Gata4 G295S^ki^* mice provides a mouse model to study human congenital heart defects. This mouse model will be of significant value to study genetic and environment modifiers for cardiac malformations along with allowing for a more mechanistic understanding of the embryologic basis of septation and valvular defects. While the dosage sensitivity of cardiac transcription factors for normal cardiac morphogenesis is generally well accepted, this mouse model demonstrates that specific mutations may have limited functional deficits. The *Gata4 G295S^ki^* mice which encodes a partially functional mutant protein, offers us a tool to define these abnormalities *in vivo*.

## Materials and Methods

### Ethics statement

Research was approved by the Institutional Animal Care and Use Committee at University of Texas Southwestern Medical Center (Protocol No. 2008-0094) and Research Institute at Nationwide Children's Hospital (Protocol No. AR09-00040) and conforms to the Guide for the Care and Use of Laboratory Animals.

### Gene-targeted mutagenesis

A strategy similar to Crispino et al., 2001 was used to generate knock-in mice harboring the *Gata4 G295S* mutation. The targeting vector contains an 8.2 kb mouse genomic fragement, which has the 2^nd^–6^th^ exons of murine *Gata4*. The construct contains a Neomycin resistance cassette flanked by loxP sites, as a positive selection marker, and HSV-tk, as a negative selection marker. Genomic DNA containing the N-terminal of the zinc finger domain of *Gata4* was subcloned into pBluescript II KS (+/−) phagemid (Stratagene). By site-directed mutagenesis, glycine at codon 295 was changed to serine (the codon GGC was changed to AGC). The targeting construct was linearized with PvuI and electroporated into 129SvES cells. Targeted clones were identified by Southern blotting. Six successful targeted ES clones identified by Southern blotting and direct sequencing for the G295S point mutation. Three clones were injected into C57BL/6 blastocysts to generate chimeric mice.

### Mouse strains and genotyping

Germline transmission was achieved by mating to C57BL/6 mice. Mice used in this study were on a mixed 129SvEv/C57BL6 genetic background. For genotyping, allelic discrimination assay was used to detect this single nucleotide change in *Gata4* locus by using fluorescent probes. This method combines PCR and mutation detection in a single step. Two TaqMan (Applied Biosystems, CA) probes were used, one for each allele. This method is implemented using the Applied Biosystems 7500Fast and TaqMan reagents to detect this point mutation in *Gata4*. The probe and primer sequences are shown in Table S2.

### Breeding and collection of mouse embryos

Mice were maintained on a 0600 to 1800h light–dark cycle, with noon of the day of observation of a vaginal plug defined as E0.5. Mice heterozygous for *Gata4 G295S* mutation were generated and genotyped as described above. Mice heterozygote for *Gata4 G295S* were mated to generate *G295S* homozygote embryos. Pregnant mothers were sacrificed at various embryonic timepoints. Littermates were used as controls for histologic sections, gene expression studies and FACS analysis.

### Echocardiographic imaging

Two dimensional and Doppler *in vivo* ultrasound images were obtained in 8 and 16 week old mice using a VisualSonics Vevo2100 imaging system (Ontario, Canada) with a mechanical transducer (MS400). Mice were anesthetized with isoflourane and echocardiograms were performed in a genotype-blinded fashion. Statistical analysis was performed using Fisher's exact test.

### Histologic section and radioactive in situ hybridization

For histological analysis, embryos and adult hearts were fixed in 4% paraformaldehyde and paraffin embedded. Hematoxylin and eosin (H and E) staining was carried out on heart sections using standard methodology. *In situ hybridization* was performed as described previously [48] using ^35^S-labeled antisense probes synthesized with T3, T7, or SP6 RNA polymerase (Maxiscript; Ambion Inc., Austin, TX) from mouse Hand1, Hand2 and Tbx5 cDNA.

### Proliferation and apoptosis assays

For the immunostaining studies, histologic sections were deparafinnized in xylene and rehydrated in phosphate buffered saline (PBS). Proliferation assays were performed using the phosphohistone H3 (PH3) antibody (Upstate Cell Signaling Solutions, Temecula, CA). The sections were permeabilized in 0.3% Triton X-100 in PBS. Sections were then blocked by 3.5% donkey serum in PBS followed by incubation with 1% rabbit anti-phosphohistone H3 antibody overnight at 4°C. Sections were then washed in PBS and Cy3 (1%) secondary antibodies (Vector Laboratories, Burlingame, CA) for 30 min. For cell proliferation studies, contiguous sections were stained for monoclonal mouse anti-human desmin using Cy3-conjugated antibody (Dako, Carpinteria, CA) to label the cardiomyocytes. The percentage of PH3-stained ventricular cardiomyocytes/total number of ventricular cardiomyocytes was calculated by analyzing a minimum of three embryos for each genotype. A minimum of four sections per embryo were analyzed, and the means and standard deviations are shown. Apoptosis (TUNEL) assays were performed using the In Situ Cell Death Detection Kit, Fluorescein (Roche) according to manufacturer instructions. Labeled ventricular cardiomyocytes were counted on a minimum of six sections of control and mutant embryonic hearts. Statistical analysis was performed using Student's *t*-test.

### Gene expression analysis

RNA was purified from embryonic hearts (E8.5–E10.5) from mutant embryos and their respective wildtype littermates using Trizol (Invitrogen). Real-time quantitative reverse transcription-polymerase chain reaction (qRT-PCR) was performed using the Taqman Universal PCR Master Mix kit (Applied Biosystems, Foster City, CA). 100 ng of total RNA was used for reverse transcription and amplification in each real-time PCR reaction using Applied Biosystems 7500 real-time PCR machine. Commercially available SYBR Green (Applied Biosystems) PCR mix was utilized for the following genes: ANF, α-MHC, cTnC, Myl3, CyclinD2, Nkx2-5, Tbx5, Mef2C, β-MHC, HNF4, Hex1, alpha-fetoprotein and Sox17. The sequences for primers are in Table S2. Mean relative gene expression was calculated from wildtype and mutant hearts after normalization to 18S ribosomal RNA, minimum of *n* = 3 per group. Statistical analysis was performed using Student's *t*-test, and a *p* value of less than 0.05 was considered significant.

### Transactivation assays

HeLa cells were transfected using Fugene 6 (Roche) according to manufacturer's instructions with 200 ng of *Gata4* wildtype and *Gata4 G295S* myc-tagged expression vectors [12], 200 ng of *Cyclin D2* pAUG-β-gal reporter vector [37]. Immunoblots were used to verify appropriate expression. Cells were cultured for 48 h after transfection, harvested and cellular extracts were prepared by sonication and normalized as described previously [49]. Chemiluminescence β-galactosidase (β-Gal) assays were performed using the luminescent β-Gal detection system (Clontech) according to the manufacturer's recommendations, and relative light units were detected using a Tropix TR717 microplate luminometer (PE Applied Biosystems).

### Flow cytometry

Embryonic heart samples for fluorescence-activated cell sorting (FACS) were prepared in the following manner: 15–20 embryonic hearts of each genotype were dissected, dissociated to a single cell solution, digested with collagenase type II (Worthington) solution, washed, spun down and resuspended in cardiomyocyte staining buffer. Cells were fixed with BD Cytofix/CytopermTM solution, permeabilized and incubated with monoclonal mouse anti-troponin T (Abcam, Cambridge, MA) and Ki67 (Abcam, Cambridge, MA ). Experiments were performed in triplicate and cells were analysed on a LSRII with DiVa software (BD Biosciences, San Jose, CA, USA).

## Supporting Information

Figure S1 Summary of M-mode echocardiographic analysis of wildtype and *Gata4 G295S^wt/ki^* mice. (A) Scatter plot showing fractional shortening in wildtype and *Gata4 G295S^ki/wt^* mice at 8 and 16 weeks of age. Scatter plots show (B) left ventricular internal diameter (LVID) at end-diastole, (C) left ventricular internal diameter during systole, (D) left ventricular anterolateral wall (LVAW) thickness at end-diastole, (E) left ventricular anterolateral wall thickness during systole, (F) left ventricular posterior wall (LVPW) thickness at end-diastole, and (G) scatter plot showing left ventricular posterior wall thickness during systole.
(JPG)

Figure S2 Patent foramen ovale, aortic valve stenosis and pulmonary valve stenosis in *Gata4 G295S^wt/ki^* murine hearts by histologic section. Interatrial communication in the form of patent foramen ovale (arrowhead in B, D) is found in *Gata4 G295S^wt/ki^* mice (B, D) as compared to wildtype littermate (A, C). (C, D) represent high magnification image of boxed area in (A, B), respectively. Thickening of aortic valve leaflets is found in *Gata4 G295S^wt/ki^* mouse (F) that had aortic valve stenosis by echocardiogram as compared to wildtype (E). Thickened pulmonary valve leaflets in *Gata4 G295S^wt/ki^* mouse are shown (H) as compared to normal leaflets in wildtype littermate (G). RA, right atrium; LA, left atrium; RV, right ventricle; LV, left ventricle; AO, aorta; PA, pulmonary artery. Scale bars indicate 200 µm.
(JPG)

Figure S3 Variable cardiac looping of *Gata4 G295S^ki/ki^* embryos during development. (A) Normal cardiac looping in wildtype E10.5 embryo as compared to incomplete looping in *Gata4 G295S^ki/ki^* in E10.5 embryo (B). Red arrow, heart. Scale bars indicate 200 µm.
(JPG)

Figure S4 Expression of Gata5 and Gata6 is unchanged in *Gata4 G295S^ki/ki^* embryonic hearts. Quantitative RT-PCR demonstrates no significant change in expression levels of Gata5 and Gata6. in E9.5 *Gata4 G295S^ki/ki^* hearts when compared to wildtype littermates.
(JPG)

Figure S5 Expression of Hand1, Tbx5 and Hand2 is unchanged in *Gata4 G295S^ki/ki^* embryos. Coronal sections through E9.5 hearts of *Gata4 G295S^wt/ki^* (A–D) and *Gata4 G295S^ki/ki^* embryos (E–H). Radioactive section in situ hybridization demonstrates mRNA expression of Hand1 (B,F), Tbx5 (C,G), and Hand2 (D,H) in *Gata4 G295S^ki/ki^* embryos is similar to *Gata4 G295S^wt/ki^* littermates. Bright-field images are shown in (A) and (E). A, atria; V, ventricle; OFT, outflow tract.
(JPG)

Figure S6 Expression of endoderm genes is not decreased in *Gata4 G295S^ki/ki^* embryos hearts. Expression of the Gata4 target endoderm genes, (A) alpha-fetoprotein and (B) Sox17 along with expression of (C) Hex1 and (D) HNF4, genes that are not Gata4 targets, is shown in E8.5 (red), E9.5 (green) and E10.5 (black) embryos. Solid bars, wildtype embryos; striped bars, *Gata4 G295S^ki/ki^* embryos.
(JPG)

Figure S7 Expression of Gata4 in *Gata4 G295S^ki/flox^*; *Nkx2-5-Cre^+^*, *Gata4 G295S^ki/flox^*; *Tie2-Cre^+^*, *Gata4 G295S^ki/flox^*; α*-MHC-Cre^+^* E10.5 embryos. Immunohistochemistry for Gata4 on histologic sections of E10.5 embryos shows decreased myocardial expression (*) and unchanged endocardial expression (arrowhead) in *Gata4 G295S^ki/flox^*; *Nkx2-5-Cre^+^* embryo (B) as compared to wildtype littermate (A). Gata4 expression is decreased in the endocardium (arrowhead) of E10.5 *Gata4 G295S^ki/flox^*; *Tie2-Cre^+^* embryo (D) compared to wildtype littermate (C). Areas of decreased myocardial expression of Gata4 (arrowhead) in *Gata4 G295S^ki/flox^*; α*-MHC-Cre^+^* E10.5 embryos (F) as compared to wildtype littermate (E). Arrowheads, endocardium;V, ventricle; scale bars indicate 200 µm.
(JPG)

Table S1 Distribution of surviving progeny at postnatal day 10 for the following mouse crosses.
(JPG)

Table S2 Primer sequences.
(JPG)
