# Supplementary figures and images for: Congenital Heart Disease–Causing Gata4 Mutation Displays Functional Deficits In Vivo
Source: PLoS Genet. 2012 May 10;8(5):e1002690. doi: 10.1371/journal.pgen.1002690 (PMC3349729; doi:10.1371/journal.pgen.1002690)

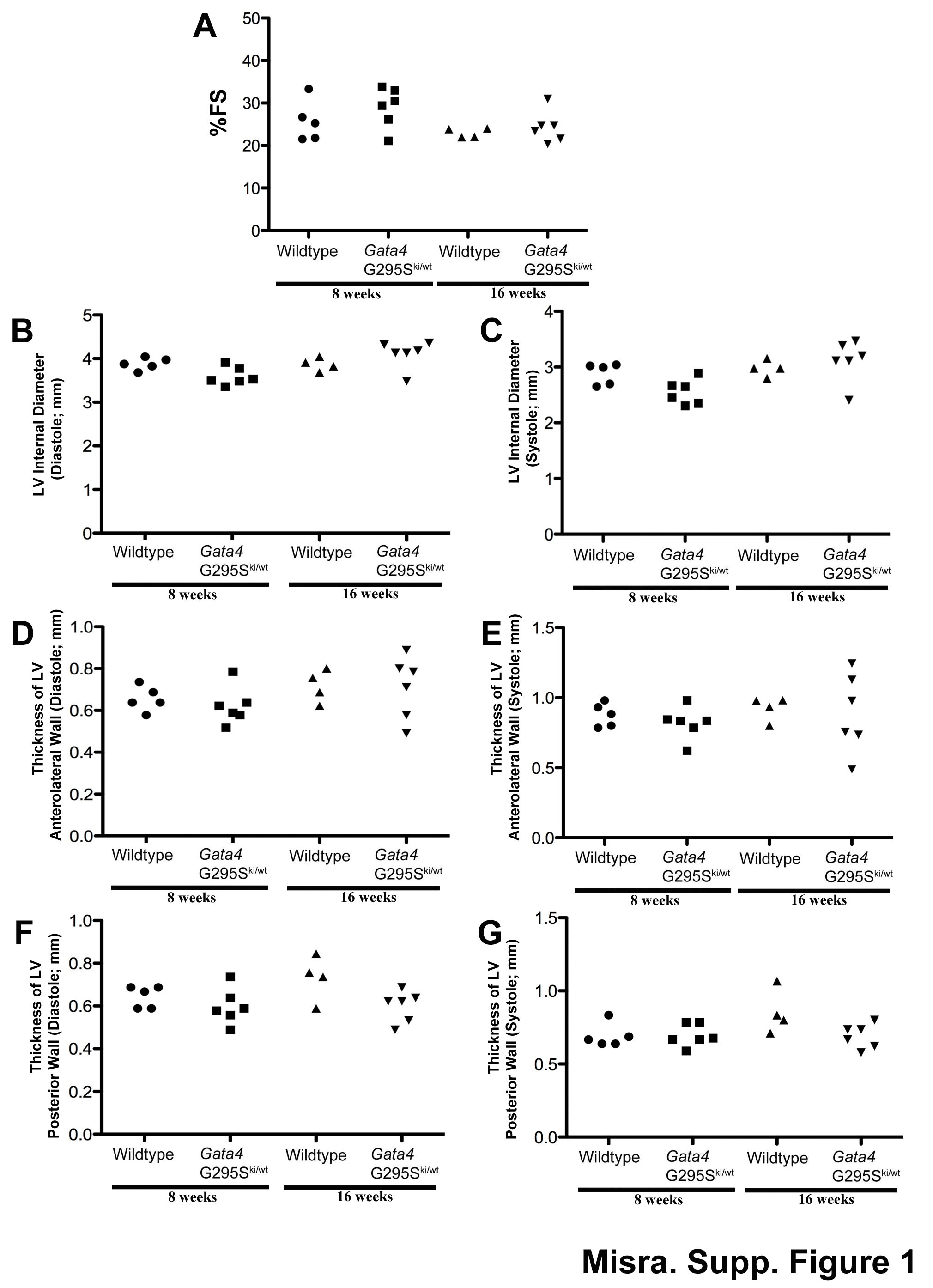

Supplement: Figure S1 — Summary of M-mode echocardiographic analysis of wildtype and Gata4 G295Swt/ki mice. (A) Scatter plot showing fractional shortening in wildtype and Gata4 G295Ski/wt mice at 8 and 16 weeks of age. Scatter plots show (B) left ventricular internal diameter (LVID) at end-diastole, (C) left ventricular internal diameter during systole, (D) left ventricular anterolateral wall (LVAW) thickness at end-diastole, (E) left ventricular anterolateral wall thickness during systole, (F) left ventricular posterior wall (LVPW) thickness at end-diastole, and (G) scatter plot showing left ventricular posterior wall thickness during systole. (JPG) [file pgen.1002690.s001.jpg]

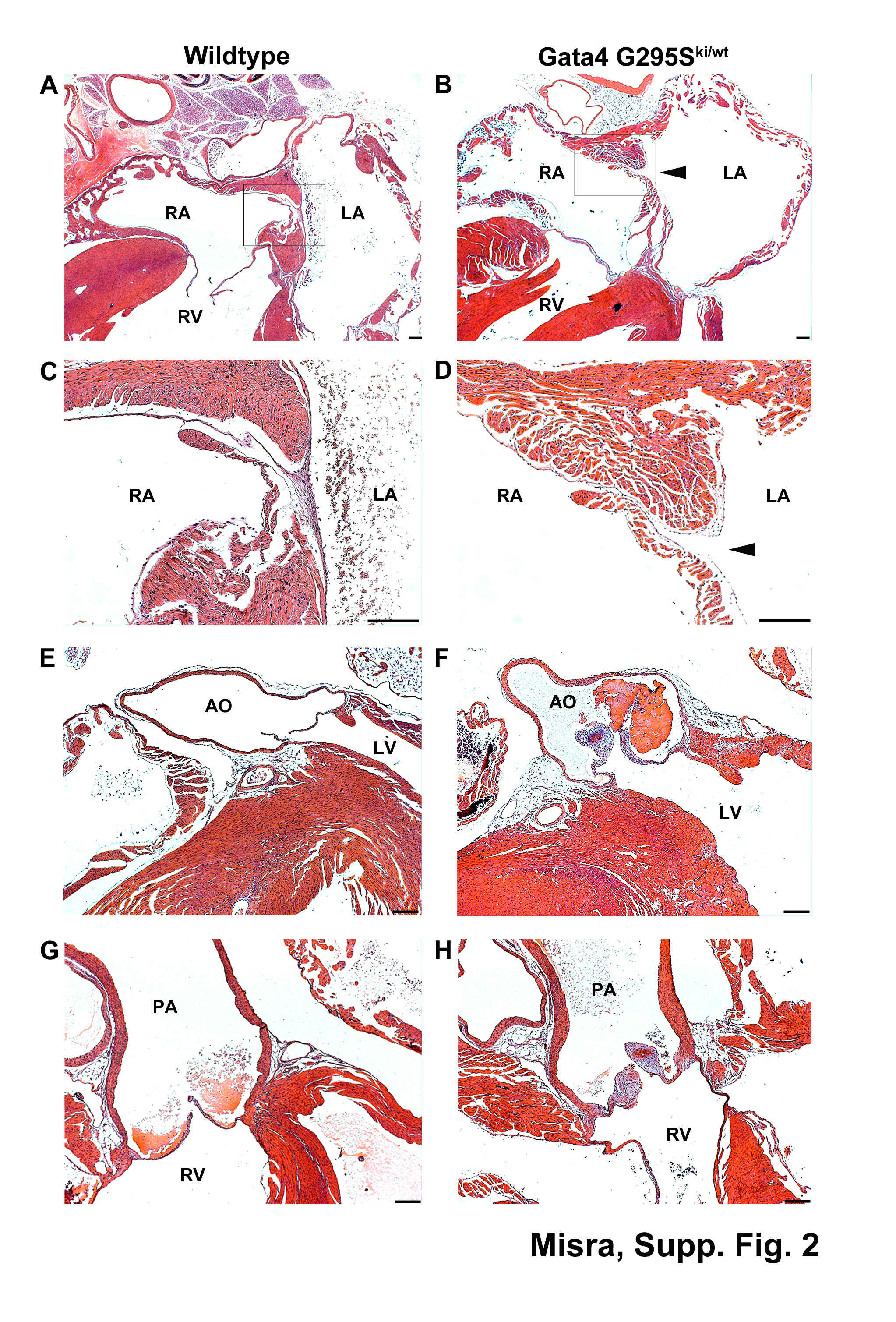

Supplement: Figure S2 — Patent foramen ovale, aortic valve stenosis and pulmonary valve stenosis in Gata4 G295Swt/ki murine hearts by histologic section. Interatrial communication in the form of patent foramen ovale (arrowhead in B, D) is found in Gata4 G295Swt/ki mice (B, D) as compared to wildtype littermate (A, C). (C, D) represent high magnification image of boxed area in (A, B), respectively. Thickening of aortic valve leaflets is found in Gata4 G295Swt/ki mouse (F) that had aortic valve stenosis by echocardiogram as compared to wildtype (E). Thickened pulmonary valve leaflets in Gata4 G295Swt/ki mouse are shown (H) as compared to normal leaflets in wildtype littermate (G). RA, right atrium; LA, left atrium; RV, right ventricle; LV, left ventricle; AO, aorta; PA, pulmonary artery. Scale bars indicate 200 µm. (JPG) [file pgen.1002690.s002.jpg]

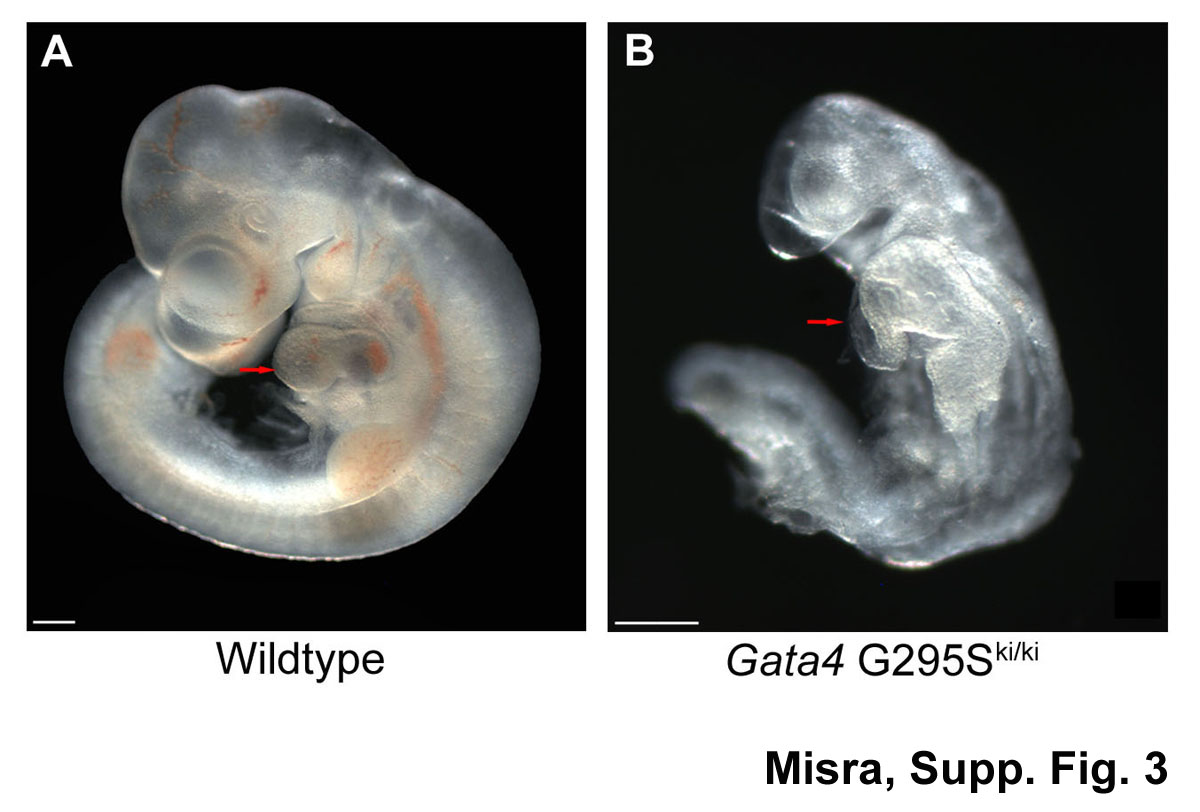

Supplement: Figure S3 — Variable cardiac looping of Gata4 G295Ski/ki embryos during development. (A) Normal cardiac looping in wildtype E10.5 embryo as compared to incomplete looping in Gata4 G295Ski/ki in E10.5 embryo (B). Red arrow, heart. Scale bars indicate 200 µm. (JPG) [file pgen.1002690.s003.jpg]

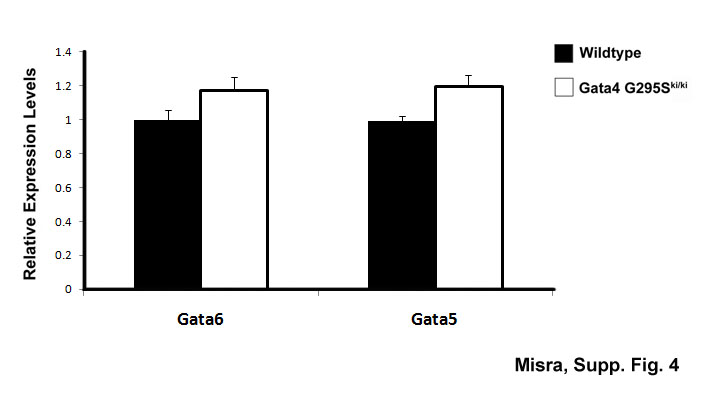

Supplement: Figure S4 — Expression of Gata5 and Gata6 is unchanged in Gata4 G295Ski/ki embryonic hearts. Quantitative RT-PCR demonstrates no significant change in expression levels of Gata5 and Gata6. in E9.5 Gata4 G295Ski/ki hearts when compared to wildtype littermates. (JPG) [file pgen.1002690.s004.jpg]

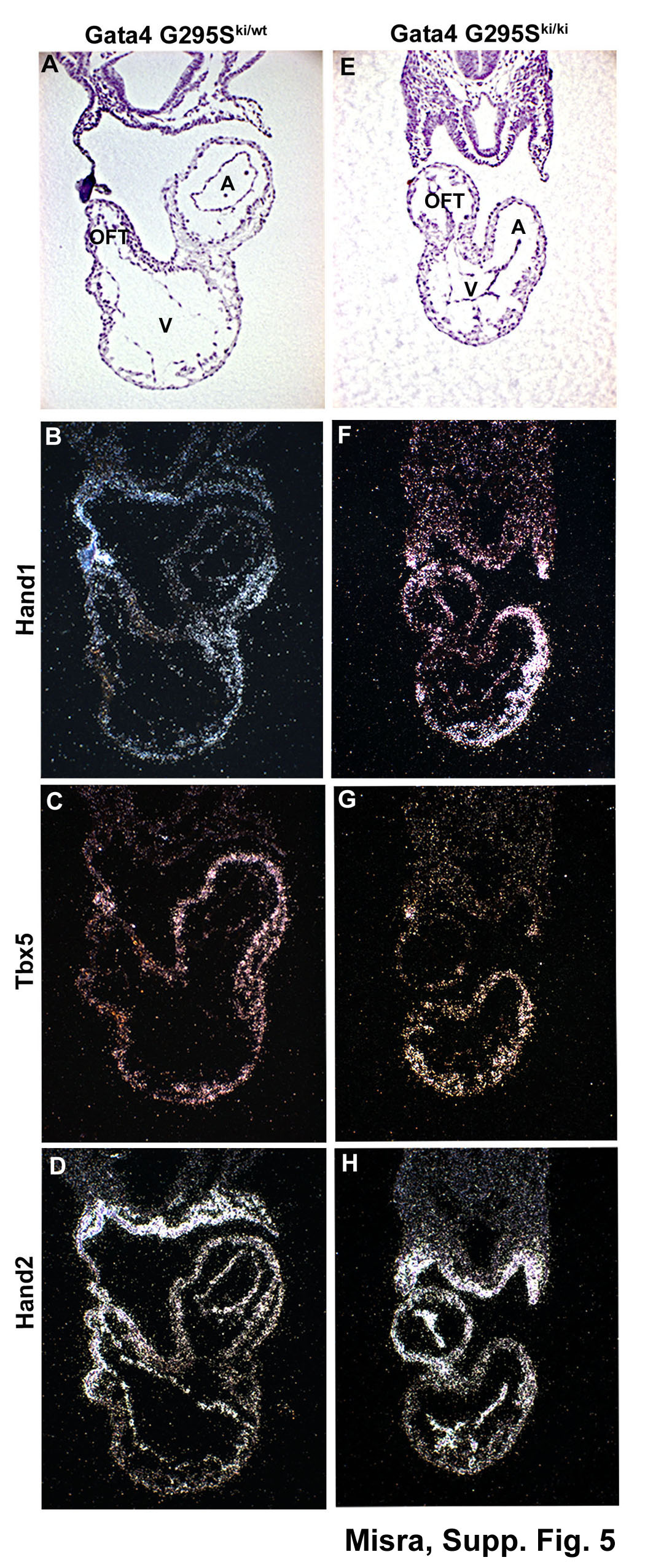

Supplement: Figure S5 — Expression of Hand1, Tbx5 and Hand2 is unchanged in Gata4 G295Ski/ki embryos. Coronal sections through E9.5 hearts of Gata4 G295Swt/ki (A–D) and Gata4 G295Ski/ki embryos (E–H). Radioactive section in situ hybridization demonstrates mRNA expression of Hand1 (B,F), Tbx5 (C,G), and Hand2 (D,H) in Gata4 G295Ski/ki embryos is similar to Gata4 G295Swt/ki littermates. Bright-field images are shown in (A) and (E). A, atria; V, ventricle; OFT, outflow tract. (JPG) [file pgen.1002690.s005.jpg]

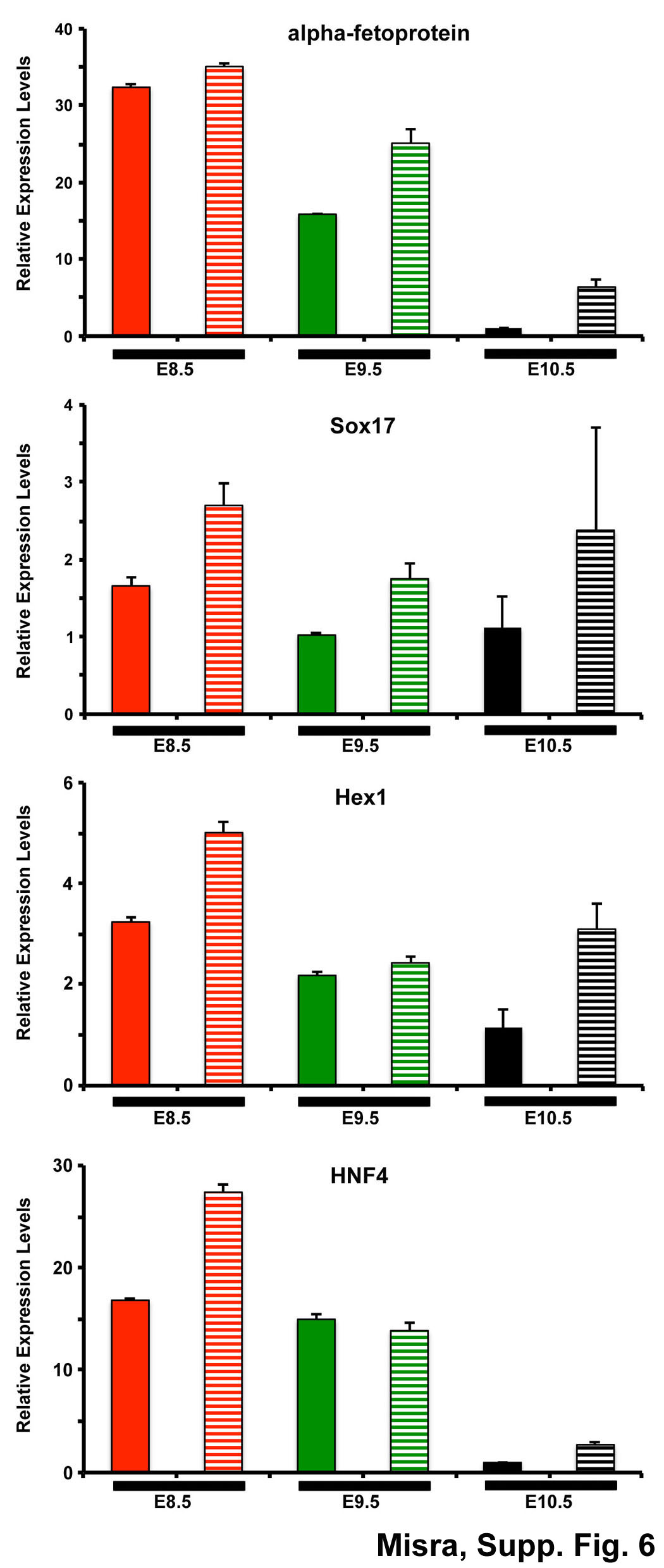

Supplement: Figure S6 — Expression of endoderm genes is not decreased in Gata4 G295Ski/ki embryos hearts. Expression of the Gata4 target endoderm genes, (A) alpha-fetoprotein and (B) Sox17 along with expression of (C) Hex1 and (D) HNF4, genes that are not Gata4 targets, is shown in E8.5 (red), E9.5 (green) and E10.5 (black) embryos. Solid bars, wildtype embryos; striped bars, Gata4 G295Ski/ki embryos. (JPG) [file pgen.1002690.s006.jpg]

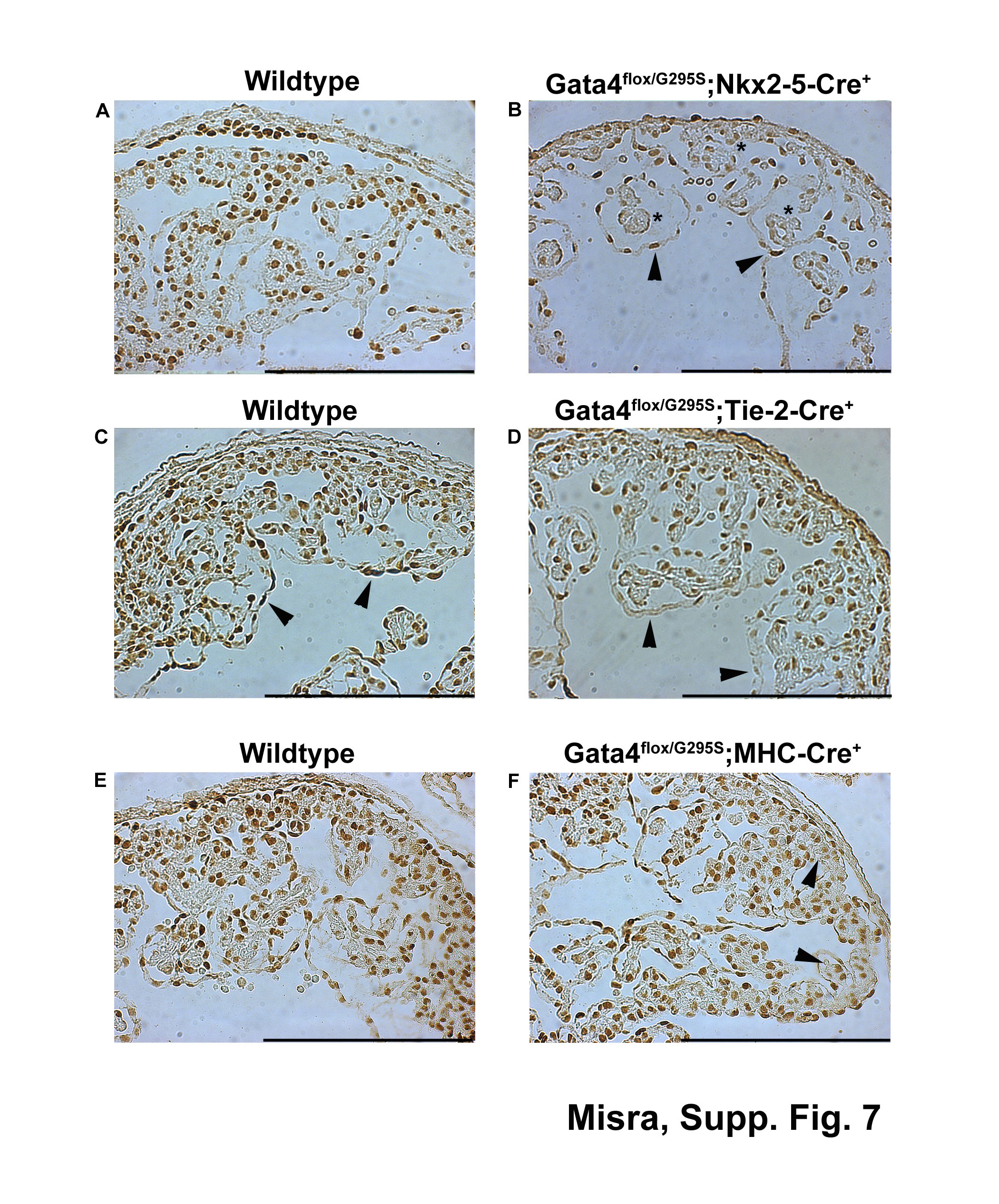

Supplement: Figure S7 — Expression of Gata4 in Gata4 G295Ski/flox; Nkx2-5-Cre+, Gata4 G295Ski/flox; Tie2-Cre+, Gata4 G295Ski/flox; α-MHC-Cre+ E10.5 embryos. Immunohistochemistry for Gata4 on histologic sections of E10.5 embryos shows decreased myocardial expression (*) and unchanged endocardial expression (arrowhead) in Gata4 G295Ski/flox; Nkx2-5-Cre+ embryo (B) as compared to wildtype littermate (A). Gata4 expression is decreased in the endocardium (arrowhead) of E10.5 Gata4 G295Ski/flox; Tie2-Cre+ embryo (D) compared to wildtype littermate (C). Areas of decreased myocardial expression of Gata4 (arrowhead) in Gata4 G295Ski/flox; α-MHC-Cre+ E10.5 embryos (F) as compared to wildtype littermate (E). Arrowheads, endocardium;V, ventricle; scale bars indicate 200 µm. (JPG) [file pgen.1002690.s007.jpg]

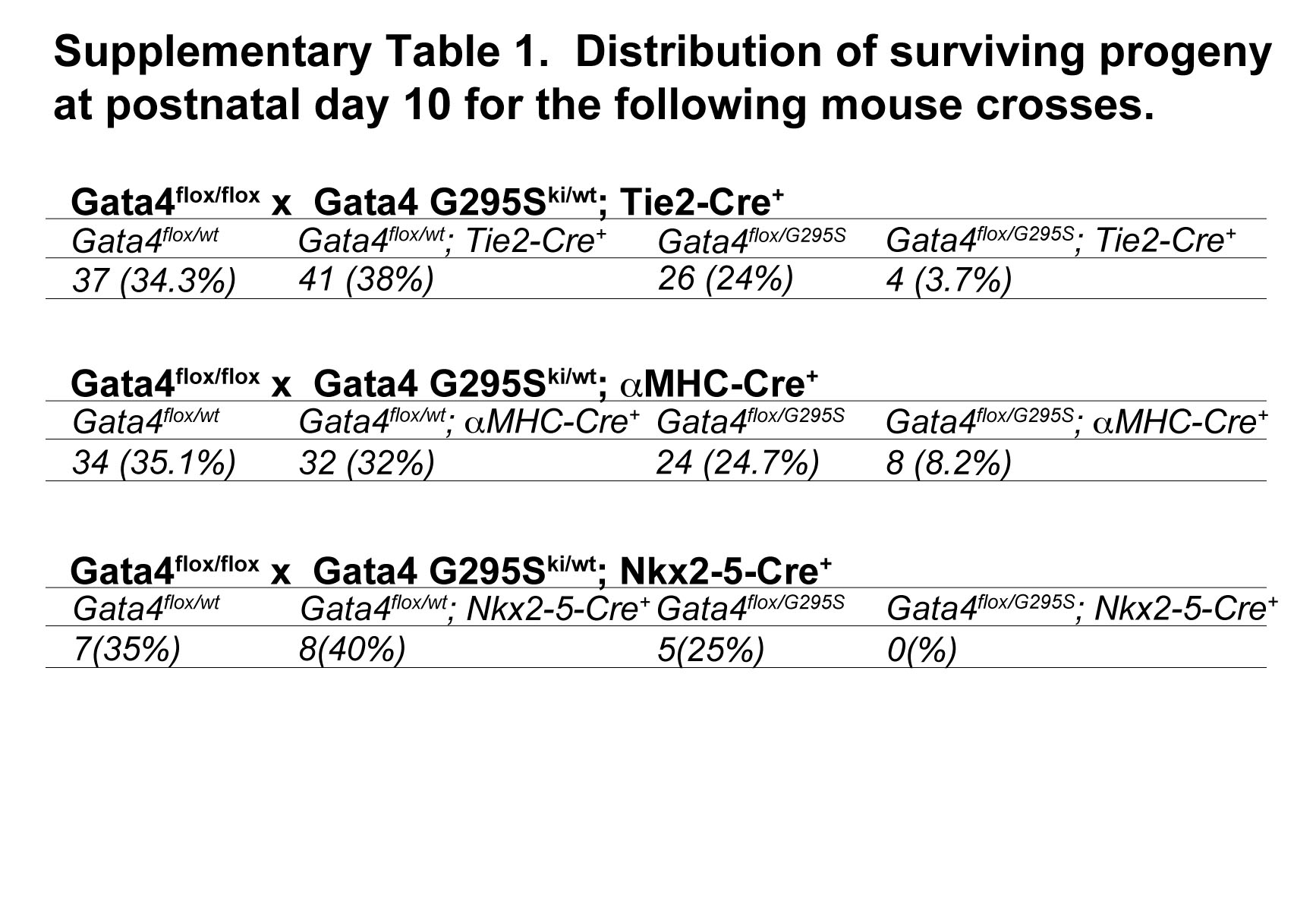

Supplement: Table S1 — Distribution of surviving progeny at postnatal day 10 for the following mouse crosses. (JPG) [file pgen.1002690.s008.jpg]

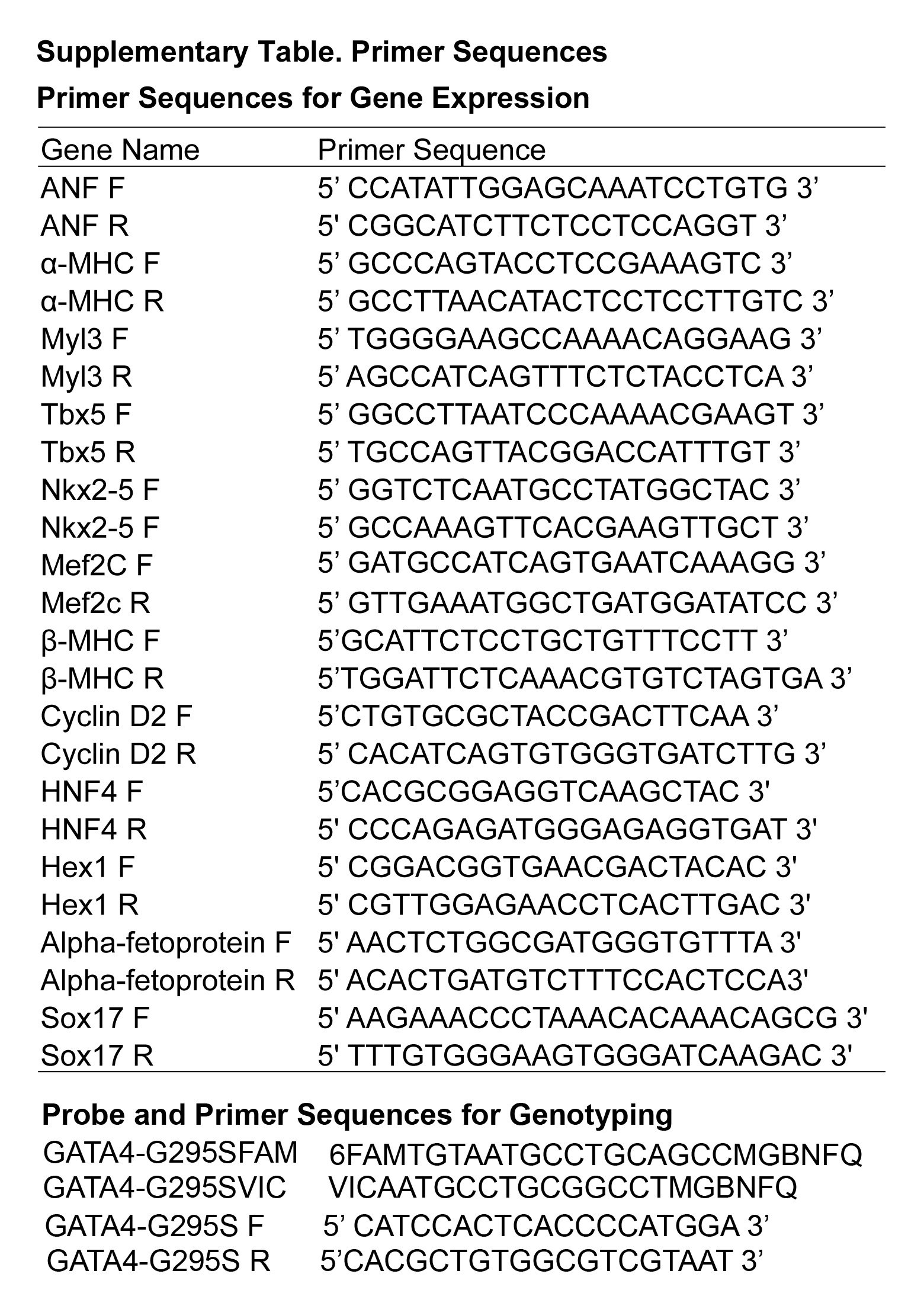

Supplement: Table S2 — Primer sequences. (JPG) [file pgen.1002690.s009.jpg]
